# Tailoring the Thermal Conductivity of Rubber Nanocomposites by Inorganic Systems: Opportunities and Challenges for Their Application in Tires Formulation

**DOI:** 10.3390/molecules26123555

**Published:** 2021-06-10

**Authors:** Lorenzo Mirizzi, Mattia Carnevale, Massimiliano D’Arienzo, Chiara Milanese, Barbara Di Credico, Silvia Mostoni, Roberto Scotti

**Affiliations:** 1 Department of Materials Science, University of Milano-Bicocca, INSTM, Via R. Cozzi 55, 20125 Milano, Italy; l.mirizzi@campus.unimib.it (L.M.); m.carnevale2@campus.unimib.it (M.C.); barbara.dicredico@unimib.it (B.D.C.); silvia.mostoni@unimib.it (S.M.); roberto.scotti@unimib.it (R.S.); 2 Department of Chemistry, University of Pavia, 27100 Pavia, Italy; chiara.milanese@unipv.it

**Keywords:** thermal conductivity, rubber nanocomposites, inorganic fillers

## Abstract

The development of effective thermally conductive rubber nanocomposites for heat management represents a tricky point for several modern technologies, ranging from electronic devices to the tire industry. Since rubber materials generally exhibit poor thermal transfer, the addition of high loadings of different carbon-based or inorganic thermally conductive fillers is mandatory to achieve satisfactory heat dissipation performance. However, this dramatically alters the mechanical behavior of the final materials, representing a real limitation to their application. Moreover, upon fillers’ incorporation into the polymer matrix, interfacial thermal resistance arises due to differences between the phonon spectra and scattering at the hybrid interface between the phases. Thus, a suitable filler functionalization is required to avoid discontinuities in the thermal transfer. In this challenging scenario, the present review aims at summarizing the most recent efforts to improve the thermal conductivity of rubber nanocomposites by exploiting, in particular, inorganic and hybrid filler systems, focusing on those that may guarantee a viable transfer of lab-scale formulations to technological applicable solutions. The intrinsic relationship among the filler’s loading, structure, morphology, and interfacial features and the heat transfer in the rubber matrix will be explored in depth, with the ambition of providing some methodological tools for a more profitable design of thermally conductive rubber nanocomposites, especially those for the formulation of tires.

## 1. Introduction

The demand for thermal conductive materials is continuously rising as a result of their wide integration in modern applications, such as aeronautics, automobiles, medical devices, photonics, optoelectronics, computer electronics, and electrical power and integrated circuits [1,2,3,4,5]. The decreasing size and the increasing power density of these devices require effective heat transfer and the need to keep temperatures within safe limits in order to achieve satisfactory reliability and performance and extend their lifecycle [1,6,7].

Therefore, the design of materials with an improved heat dissipation ability to prevent degradation caused by overheating represents a crucial issue for better thermal management, especially in terms of cost-effective and efficient industrial applications.

Owing to their light weight, ease of processing, design freedom, and cost-efficiency, recent emerging technologies benefit from the employ of polymer-based thermal conductive materials as flexible alternatives to the traditionally applied metals [1,8,9,10,11,12,13,14]. In fact, though metals display a very high thermal conductivity (λ), they exhibit critical drawbacks, such as high density, corrosion and oxidation tendencies, and limited design possibilities [15].

However, polymers and polymer composites intrinsically possess low λ values due to their relatively low atomic density, weak interactions and chemical bonding, complex molecular structure, and high anharmonicity in their molecular vibrations [16,17,18]. These characteristics severely restrict their industrial application when fast and effective heat dissipation is required, such as in the thermal management of electronics [2].

Over the past few decades, considerable endeavors have been devoted to the design and fabrication of polymer composites with higher λ values, mainly by exploiting thermally conductive fillers or fabricating special composite microstructures [1,9,10,11,16,18,19,20]. In particular, in the field of electronic packing, thermal interface materials (TIMs) fabricated by adding highly thermally conductive fillers to polymers constitute an important branch of advanced thermal management materials [1]. Both ceramic materials, including silicon carbide (SiC) [21,22], alumina (Al_2_O_3_) [23,24], boron nitride (BN) [25], zinc oxide (ZnO) [26,27,28], and aluminum nitride (AlN) [29,30], and thermally conducting lightweight carbon derivatives, such as carbon nanotubes (CNTs) [31], carbon blacks (CBs) [32], carbon fibers (CFs) [33], graphite [34,35], and reduced graphene oxide (rGO) [36,37,38], have been widely used to impart superior thermal conductivity properties to several polymers. In particular, high-aspect-ratio fillers, such as whiskers and platelets, have been studied since they can assemble even at a lower loading, forming continuous thermally conductive pathways in the polymer matrix and enhancing thermal transfer [39]. In addition, it has also been demonstrated that the construction of three-dimensional (3D) thermally conductive networks and the incorporation of filler with a different chemical nature and dimensionality (i.e., hybrid fillers) represent key factors for improving the λ of the composites [40,41,42,43].

Interfacial thermal resistance arises upon filler incorporation due to differences between the phonon spectra and scattering at the hybrid interface between the different phases, resulting in a drop in λ [20]. Thus, tailored filler functionalization strategies are essential to avoid discontinuities in the thermal conductivity in the final composite material [44,45,46,47].

Moreover, the most recent studies report that a significant improvement in λ can be achieved only when high filler loadings are included in the polymer matrix [48,49]. Although this leads to the generation of an effective thermally conductive pathway, it dramatically depresses the mechanical behavior and the cross-linking density of the composites [11].

These issues become even more decisive for strategic materials such as rubber composites, a very significant category of polymeric materials with numerous applications in miniaturized systems, such as cable accessories, gate dielectrics, electronic devices, gas barrier materials, and advanced binders for energy storage devices, where an optimal balance between efficient thermal management and operational efficiency has continued to constitute a challenge [50,51].

Yet, rubber composites are also widely utilized in large-scale products. One of the most common products is tires, which usually work under dynamic service conditions and whose heat dissipation is tightly connected with personal safety [52,53,54,55]. In fact, in continuous operation, tires experience intense low-frequency mechanical deformations arising from the internal friction at the molecular level between filler and filler, filler and rubber, and rubber and rubber, resulting in a significant heat build-up [54]. Because of the low thermal conductivity of elastomers, in line with most polymers, the heat generated is accumulated, leading to a high local temperature that may reach 120 °C or higher. This high internal overheating is the main reason for the failure of the tread base and shoulder of tires, which influences the service life of tires, especially truck and bus radial tires, which become more sensitive to ruptures, cuts, and delamination [54].

In general, the identification and mitigation of tire overheating effects require experimental studies on: (i) the thermal behavior under the specific operational conditions; and (ii) the basic mechanisms of heat dissipation in the tire in use. In addition, an in-depth investigation of the influence of the rubber formulation and rubber compounding on the heat dissipation properties is mandatory [54,55]. However, there are few literature reports dealing with the tailoring of the thermal conductivity of rubber composites utilized for tire production.

This background unveils the high potentiality in terms of long-term commercial fallouts that can be gathered by the tailoring of the thermal conductivity of rubber composites through the controlled design of their structural elements, i.e., the filler, the polymer matrix, and their interactions, which determine the heat generation and withdrawal into the surrounding space.

Bearing these challenges in mind, this review briefly presents the most used fillers for tuning the thermal conductivity of rubber composites, focusing on inorganic and hybrid systems, which may provide a realistic upgrade of the properties and a transfer of lab-scale formulations to technological applicable solutions.

In detail, a brief tutorial on the thermal conductivity mechanism in polymer composites along with a short description of the main parameters influencing the λ value will first be supplied. The focus will then be oriented toward thermally conductive rubber nanocomposites. In this context, and aiming to suggest implementations, a survey of different conductive fillers utilized in rubber nanocomposites will be carried out, with specific attention paid to ceramic systems such as Al_2_O_3_, BN, and ZnO, which represent promising candidates in terms of performance, availability, and costs. The effects of their loading, structure, morphology, surface functionalization, and combination in hybrid fillers on the generation of thermally conductive pathways in the rubber matrix will be described.

Specific insights into their exploitation in the design and development of advanced thermally conductive rubber nanocomposites utilized for tire formulation will also be delivered.

## 2. Thermal Transport in Polymer Composites: Basic Principles and Parameters Affecting the Thermal Conductivity

Thermal conductivity is a physical quantity that measures the capability of a substance to conduct heat when heat transport by convention and irradiation mechanisms is negligible. Thermal conductivity entails a non-uniform distribution of the internal temperature in the object and normally is expressed by Fourier’s law (1):

(1) $$q=\lambda A\;\frac{\Delta T}{L}$$

where *q* is the rate of heat conduction (W); *λ* the thermal conductivity (Wm^−1^K^−1^); *A* the cross-sectional area for the heat transfer (m^2^); Δ*T* the temperature difference (K); and *L* the length of the conduction path (m).

Depending on the material families, heat conduction in solids can be mediated by: (i) vibrations and collisions of molecules, (ii) propagation and collisions of phonons, or (iii) diffusion and collisions of free electrons.

A phonon is the quantum mechanical description of a normal mode of vibration, an elementary vibrational motion in which in a lattice of atoms or molecules uniformly oscillates at a single frequency. While normal modes are wave-like phenomena in classical mechanics, phonons also have particle-like properties in a way related to the wave–particle duality of quantum mechanics [56].

Generally, in metals heat is transferred almost exclusively by electrons, while in crystal and amorphous materials, phonons are identified as the main heat carriers.

In fact, in the ordered structure of crystals, it can be assumed that, when a lattice atom vibrates, it transfers vibrational energy to the adjacent atoms that start to oscillate, which then propagates the energy further into the sample. According to this model, the presence of interfaces, defects, or any discontinuity in the crystalline structure, such as point defects, dislocations, grain boundaries, and many other types of defects, can drastically decrease the thermal conductivity due to phonon scattering [57].

Regarding amorphous materials and, in particular, polymers, they can be thermally compared to a combination of many defects that slow down the heat transfer. In fact, though in the crystalline regions of polymers heat transfers rapidly due to the ordered molecular chains where atoms can vibrate slightly near the equilibrium position, the recurrent presence of random entanglement leads to the scattering of phonons and affects phonon transport, resulting in low *λ* values [20].

This complex situation has been described by a mathematical model that approximates the thermal conductivity of polymer-based materials by the equation [58,59]:

(2) $$\lambda =C_{p}\upsilon l/3$$

where *C_p_* is the specific heat capacity, υ  is the speed of sound, and *l* represents the phonon mean free path, which assumes very low values for most polymers because of the scattering with other phonons, defects, and grain boundaries.

Therefore, most polymers have low λ values, ranging between 0.2 and 0.5 Wm^−1^K^−1^, which make them poorly exploitable for applications that require remarkable heat conduction. However, polymers have a number of advantageous features, such as light weight, enhanced processability, hindered water absorption, high electrical resistivity, high voltage breakdown strength, resistance to corrosion, and, most significantly, low cost. The thermal conductivity of polymers can be upgraded by the incorporation of highly thermally conductive fillers, thus developing polymer composites. In these materials, the thermal management relies on the thermal conductivities of both the polymer matrix and fillers, on the morphology of composites, and on the interaction at the interface between the polymer and the filler.

**Figure 1:**
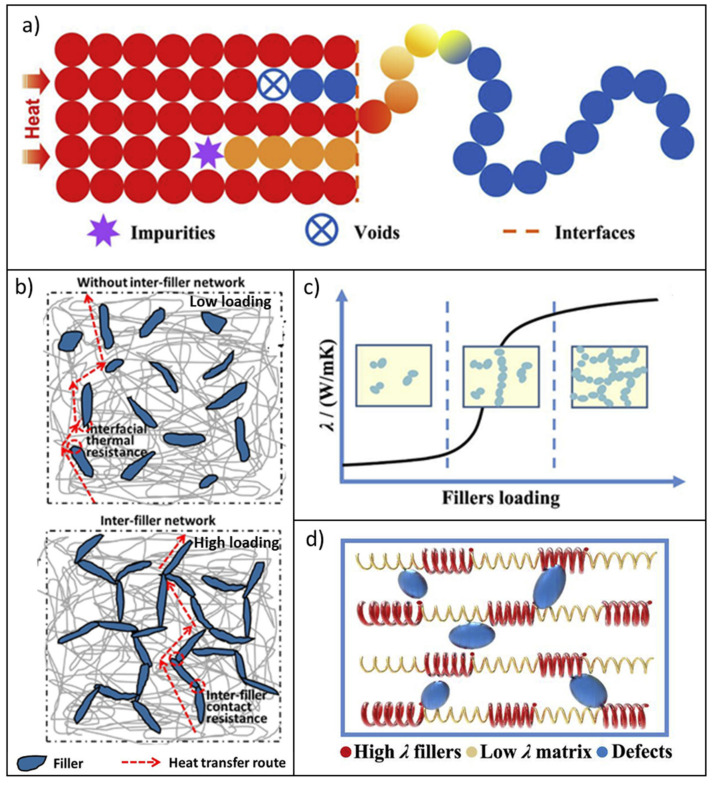
(**a**) Mechanism of heat transport in polymer composites; (**b**) generation of thermally conductive pathways in polymer composites as a function of filler loading; schematic diagrams of (**c**) percolation and (**d**) thermoelastic coefficient theories that describe the thermal conductivity in polymer composites. Adapted from [14] (© 2021 Elsevier Ltd. All rights reserved.) and [16] (© 2021 Elsevier B.V. All rights reserved.).

To predict and analyze the λ of polymer composites, several theoretical models have been proposed. In general, they supply equations that connect the thermal conductivity of the composites to those of the polymer matrix and filler and to the filler volume fraction (e.g., the Maxwell–Eucken and Bruggerman models) [11,18]. However, these approaches provide good agreement with the experimental data only for spherical fillers and assume an ideal filler–matrix interface, i.e., no phonon scattering phenomena and thermal resistance at the interface [60]. The interface actually plays a key role in determining the effective λ value of polymer composites. It can be assumed that, though filler particles are surrounded by polymer chains in a composite, only smaller parts of those chains are readily in contact with the fillers. This situation leads to a high degree of phonon scattering and, consequently, thermal resistance at the interface (Figure 1a) [20,60].

Many attempts have recently been made in order to consider the interfacial thermal resistance and the influence of fillers with different morphological features (e.g., aligned continuous fibers, layered materials, and heterogeneous and misoriented ellipsoidal particles), offering the possibility to retrieve more accurately the λ of the composites [18]. In this context, the acoustic mismatch model (AMM) and the diffuse mismatch model (DMM) have conventionally been exploited for calculating the thermal boundary conductance of interfaces [18]. In the AMM, the phonon transfer mechanism through the interface is assimilated to the behavior of acoustic waves. Thus, the transmission coefficient and, in turn, the interfacial thermal resistance are dependent on the density of the materials in contact and on the group velocity of the longitudinal phonons in the two media. However, the AMM works properly only at very low temperatures and does not take into account the phonon scattering at interfaces.

The scattering phenomena are instead considered in the DMM, where the incident phonons are assumed to lose the memory of their original state and scatter diffusively and elastically in the two media. This model also gives good results for higher temperatures (T > 15 K) but overestimates the interfacial thermal resistance for materials that possess a large mismatch in the phonon density of states since inelastic phonon scattering is not considered [18,61].

In addition to these theories, several other models have been proposed for explaining thermal conduction in polymer composites, including thermal conduction path, thermal percolation, and thermoelastic coefficient models (Figure 1b–d) [18,62,63,64,65].

In the thermal conduction path theory [14], the generation of a continuous conductive network of thermally conductive fillers grants the heat transfer within the composite. According to this theory, when the filler loading is low, “sea-island” systems, i.e., isolated filler particles or aggregates surrounded by a polymer matrix with a low *λ* value, occur. In these conditions, the polymer matrix limits the thermal conduction channels, leading to a negligible shift in the *λ* value of the composite. Increasing the filler volume fraction enhances the formation of thermally conductive networks and, consequently, achieves effective thermal transport (Figure 1b).

Connected to the previous model, the possibility to describe the thermal transport in polymer composites by referring to a percolation theory remains controversial (Figure 1c). In fact, though high filler volume fractions provide interconnected clusters inside the polymer matrix and increase the λ value, no abrupt changes in this value as a function of the filler loadings can generally be detected. Thus, a thermal percolation threshold, similar to the electrical one, becomes hard to define. This effect may be correlated to the difference in the vibrational modes of the filler and the matrix, which make the phonon scattering severe and hinder the heat transfer. However, some studies report the occurrence of percolation phenomena for composites enclosing fillers with particularly high *λ* values. Although thermal percolation seems to be achieved, it remains an open issue to determine the relative contribution of thermal transport occurring via the filler network or via the polymer matrix [14,18].

Finally, the thermoelastic coefficient theory equates the *λ* to the thermoelastic coefficient of the phonon propagation. According to this model, *λ* becomes a macroscopic property depending on the combined action of highly thermally conductive (and thermoelastic) fillers and the (thermoelastic) polymer matrix with low thermal conductivity, which are closely in contact and share interfaces in the composite (Figure 1d). Similar to the interference phenomenon that undergoes vibration and waves at the interfaces between two phases with different elastic coefficients, the phonons will also scatter and hinder the thermal conduction. Thus, the improvement in the λ value of the composite is in accordance with the combined enhancement mechanism of the thermoelastic coefficient of the whole system and occurs gradually with an increasing loading of the thermally conductive fillers, without a sharp increase [9,14,18].

**Figure 2:**
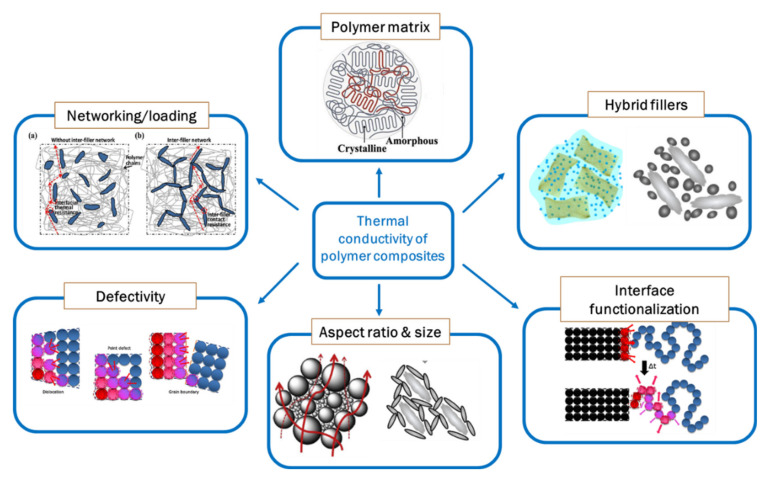
Main factors influencing the thermal conductivity in polymer nanocomposites.

Based on the above-described theories, laboratory practices showed that the effective thermal conductivity of a polymer composite is a function of the *λ* of the constituents and, in particular, of the filler volume fraction, the particle shape and size, the particle dispersion, and their surface functionalization (Figure 2).

Of course, the polymer matrix plays a crucial role in governing the thermal conductivity of the composite. In fact, random entanglement of the polymer chains and high values and polydispersion of their molecular weight induce low-crystallinity features. Moreover, inharmonic vibrations due to low regularity or branching of macromolecule chains [66,67,68,69], interfaces and defects, and polymers with complex structures (e.g., crystallization, orientation, and micro-scale ordered structures) foster phonon scattering [16].

As already mentioned, the volume fraction of thermally conductive fillers plays a key role in determining the final λ value of the composite. In general, high loadings are needed to form thermally conductive pathways and to achieve high *λ* values, inevitably depressing the mechanical and processing performances of the composite and raising their fabrication costs [11,70]. Moreover, as cited before, it appears to be difficult to define a clear percolation threshold in thermally conductive composites, even if some studies report *λ* values that first increase and then decrease upon a determined filler volume fraction being reached, claiming that filler agglomeration phenomena occur that destroy the thermal conduction paths [71,72].

Commonly, to reach high λ values, significant amounts of fillers are enclosed in the polymer matrix, with consequent damage to the mechanical and processing properties of the composite. Moreover, as the fillers are usually more expensive than polymers, the final costs would increase. Thus, further efforts are needed to obtain efficient thermal transport in the composite with a lower filler volume fraction.

Filler size is also critical for tuning the thermal conductivity of polymer composites. Large fillers provide a lower filler/polymer interface, thus suppressing the interfacial thermal resistance and increasing λ [73,74,75,76,77]. Although thermal transport through larger particles is easier than through smaller ones, when the filler size is in the nanometric range, filler properties can also promote an increase in λ. For example, the melting point of nanometallic particles is generally significantly lower than that in their bulk state; this effect may result in easier sintering of the nanoparticles during the polymer curing process, with the formation of a heat conductive network. Hence, for a single filler, the choice of the size will depend on the nature of the filler and the balance between its ability to form particle chains and reduce phonon scattering at its surface.

Increasing the filler’s polydispersity can facilitate the assembly of thermal conductive pathways since smaller fillers may form a conductive bridge between large filler particles [78]. This is also the case for the so-called “hybrid fillers”, i.e., a combination of thermally conductive fillers with different sizes, shapes, and chemical natures (vide infra), which have been demonstrated to impart better dispersion and effective thermal conduction paths in the polymer matrix [79,80,81].

Thermally conductive particles can be supplied by or prepared with different isotropic and anisotropic shapes, such as spheres, polyhedron, fibers, whiskers, and platelets, whose assembly and interfacial interaction with the polymer chains remarkably affect the λ value of the composites. For instance, 1D and 2D fillers with a high aspect ratio can form more continuous thermally conductive pathways in the polymer matrix, even at a relatively low volume fraction, compared with 3D fillers [10,14]. However, spherical fillers generally lead to isotropic thermal conductivity within the nanocomposites, while anisotropic *λ* values, depending on the filler orientation, are retrieved for anisotropic particles. This depicts a delicate point, since peculiar properties can be hindered or enhanced as a function of the filler’s aspect ratio and alignment. Therefore, a trade-off between the ability to develop thermally conductive networks and the generation of interfacial thermal resistance, due to inhomogeneous or ineffective filler–filler and filler–polymer interactions, has to be achieved.

The interfacial thermal resistance in polymer composites originates generally from a mismatch in the surface conditions of the two phases in contact (i.e., thermal contact resistance) or from a difference in the phonon spectra between the filler and the polymer matrix [82]. A significant reduction in the thermal resistance at the interface can be accomplished by filler surface functionalization. Different strategies have been proposed, such as exploiting surfactants [83] and silane coupling agents [40,84,85,86,87,88,89,90,91,92,93,94,95,96,97,98,99,100,101], grafting functional polymers [102,103,104,105], and generating core–shell structures [106,107,108,109,110,111,112,113,114,115,116,117,118,119]. The common target of these approaches is an increase in the adhesion between the filler and the polymer and a reduction in the amount of interfacial phonon scattering. It has to be mentioned that surface functionalization should in principle improve the dispersion of fillers in the polymer matrix, reducing the viscosity during the composite’s processing.

However, surface modification may also induce the generation of defects, which have a detrimental influence on the phonon transport. In fact, the presence of point (vacancy and interstitial atoms), line (dislocations), surface (grain boundary and phase interfaces), and body (cavities and bubbles) defects result in phonon scattering phenomena [120,121,122,123,124,125], which may impact on the λ value of the composite. For instance, surface oxidation of CNTs in strongly acidic conditions on the one hand improves their dispersion in the polymer matrix; on the other hand, it creates defects at the surface that damage the filler structure, reducing its intrinsic thermal conductivity [126,127].

In summary, mastering the models and the main factors affecting the thermal transport properties of polymer composites may help to select the most promising composition and conditions for the design of effective thermally conductive composites. This is a challenging point, especially for rubber nanocomposites, where a balance of many other properties, such as mechanical, thermal, and electrical insulation and dissipation features, has to be attained. All these aspects will be described and commented on in the following sections by referring to filler systems utilized for the development of thermally conductive rubber compounds, with specific insights for those applied in tire technology.

## 3. Filler Systems for Improving the Thermal Conductivity of Rubber Nanocomposites: The Role of Inorganic Nanomaterials

Rubber thermoset elastomers are natural rubbers or synthetic polymer macromolecules composed of partially entangled molecular chains, whose cross-links are generated during vulcanization or curing reactions. They include for instance natural rubber (NR), styrene butadiene rubber (SBR), nitrile butadiene rubber (NBR), and silicone rubber, which are basically insulators, due to their inherently poor thermal conductivity ranging between 0.1 and 0.2 W m^−1^ K^−1^ [10]. This represents a significant drawback for their applications, since the accumulated internal heat will accelerate their aging and deterioration [68,69]. The incorporation of thermally conductive fillers (Table 1) is the most common approach to overcoming this barrier for the efficient technological application of rubber polymers.

The following sub-sections will summarize the most recent attempts to improve the thermal conductivity of rubber nanocomposites by exploiting, in particular, inorganic (Al_2_O_3_, BN, and ZnO) and hybrid carbon-based/ceramic fillers, with a special emphasis on the examples oriented toward technological applicable solutions, such as rubber products for tires.

### 3.1. Carbon-Based Fillers

Usually, carbon-based fillers, such as graphite, CNTs, graphene, and carbon fibers, are utilized for imparting a satisfactory heat transfer ability to rubber polymers, owing to their high thermal conductivity (100–4000 W m^−1^ K^−1^) along the in-plane direction [11]. While on the one hand this has represented an opportunity to produce a highly conductive composite by filler alignment, on the other hand these kinds of composites are not suitable for some applications where anisotropic thermally conductive composites are needed, such as tires. Moreover, though carbonaceous fillers appear to be effective in the upgrade of the thermal transport, their consistent volume fraction in the matrix may sacrifice the composite’s processability, inducing delamination or peeling phenomena that damage the composites.

CNTs have been exploited to produce various thermally conductive rubber nanocomposites [133,134,135,136]. Despite the promising thermal transport properties imparted by these materials, their utilization still presents issues to resolve, such as obtaining well-structured and uniform CNTs with low defectivity, decreasing the high thermal resistance at the interface between the filler and the rubber matrix [137], improving the disentanglement of their bundles that hinder their dispersion [138], and reducing the loading required to produce a conspicuous improvement in λ, which also affects the cost of the nanocomposite’s production [139]. It has to be mentioned also that, since CNTs are highly electrically conductive, their use in a formulation for electrical insulation could be complicated.

The utilization of graphene in rubber nanocomposites has prompted much research effort due to the unique properties of this 2D material, namely the zero-gap band structure, high electron mobility, and high thermal conductivity [140]. Although reviews describing the thermal management in polymer composites are widely present in the literature [1,8,9,10,11,12,13,14,16,18,20], studies focused on the thermal conductivity of graphene–rubber composites have only recently arisen [62,141,142,143,144,145]. The main issues with the fabrication of rubber/graphene nanocomposites are related to the homogeneous dispersion of graphene, the improvement of the interfacial interaction with the rubber matrix, and its effect on the vulcanization process. Moreover, the production of this material is limited by low yields. As a consequence, graphene derivates, such as GO and rGO, generally replace pristine graphene in the applications due to the higher yield of their production processes and their similarity to neat graphene [146].

Yin et al. [141] analyzed the effect of GO incorporation on the thermal and mechanical properties of SBR. GO contains polar groups that in non-polar rubber lead to a poor filler dispersion in the matrix, causing a reduction in both thermal transport and mechanical properties. To enhance the compatibility between the filler and the matrix, the authors utilized a non-covalent functionalization of the GO with polyvinylpyrrolidone (PGO), which is an amphiphilic polymer. The thermal conductivity of SBR nanocomposites enclosing 5 parts per hundred rubber (phr) of PGO was significantly higher compared with that of the composites containing the same fraction of GO. This was explained by referring to the reduction in the acoustic phonon impedance caused by GO functionalization, which produces a better adhesion of the filler to the rubber matrix and generates tortuous paths improving the blend’s resistance to solvent penetration and heat transfer. In addition, the SBR/PGO showed significantly higher tensile and tear strengths compared with those of pristine SBR.

The possibility of tuning the surface functionalization and the recent attempts to combine these carbon-based materials with other thermally conductive fillers are opening new avenues for their employ in the tire industry (see Section 4).

Graphite-based fillers may be a cost-effective conductive alternative to CNTs and graphene.

Mu and Feng [147] explored the thermal conductivity of silicone rubber compounds filled with different amounts of expanded graphite (EG), fabricated by melt mixing or solution intercalation approaches.

**Figure 3:**
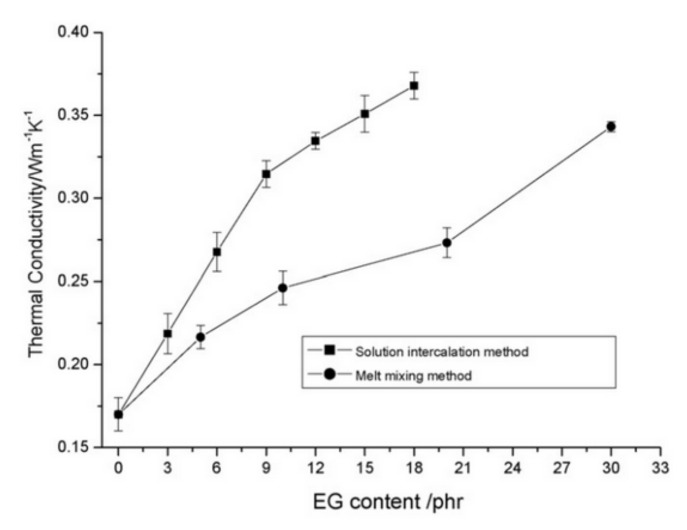
Thermal conductivity vs. fraction of EG for silicone rubber/EG composites prepared by different methods [147]. Copyright © 2021 Elsevier B.V. All rights reserved.

**Figure 4:**
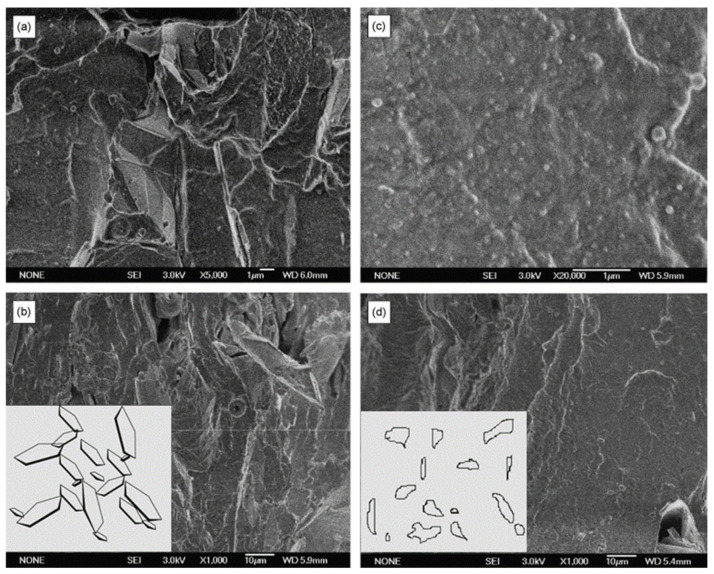
SEM micrographs of silicone/EG (10 phr) composites prepared by solution intercalation (**a**–**c**) and melt mixing (**d**). Insets in (**b**,**d**) represent the structural models proposed, showing the different surface-to-volume ratios of EG particles and the formation of conducting paths in the silicone rubber matrix. Adapted from [147]. Copyright © 2021 Elsevier B.V. All rights reserved.

Nanocomposites obtained by solution intercalation displayed much higher λ values compared with those prepared by the traditional melt mixing process (Figure 3). This was explained by Scanning Electron Microscopy (SEM) studies performed on the nanocomposites, which revealed that EG, dispersed in the silicone rubber matrix, shows a different surface-to-volume ratio depending on the compounding method (Figure 4).

In detail, EG particles with a large surface-to-volume ratio and forming a conductive network are clearly detectable when the filler is introduced into the polymer by solution intercalation. Therefore, even low filler loadings (i.e., 9 phr) produced a significant improvement in the thermal conductivity. Conversely, by melt mixing, pronounced changes in the internal EG structure were observed, in conjunction with a remarkable reduction in the surface-to-volume ratio of EG fillers. Consequently, only at higher EG contents can thermally conductive paths be generated. These results underline the importance of the anisotropy and aspect ratio of conductive fillers in determining the improvement to the thermal conductivity of rubber composites.

**Figure 5:**
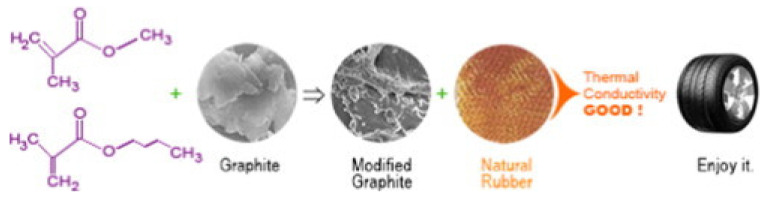
Scheme of the graphite’s modification with polyacrylates and introduction into NR for the generation of rubber composites for tire applications [148]. Copyright © 2021 Elsevier B.V. All rights reserved.

Song et al. [148] explored the possibility of exploiting graphite to improve the heat dissipation properties of rubber composites for tire applications. To this aim, graphite was functionalized at the surface with polyacrylates deriving from the emulsion polymerization of methyl methacrylate, *n*-butyl acrylate, and acrylic acid. Eight different samples were prepared as a function of the ratios between the filler and the acrylate monomers and between the hard and soft monomers (labelled as 1#–8#). Subsequently, bare (sample 0#) and modified graphite particles were introduced into NR with a loading of 30 phr (Figure 5).

**Figure 6:**
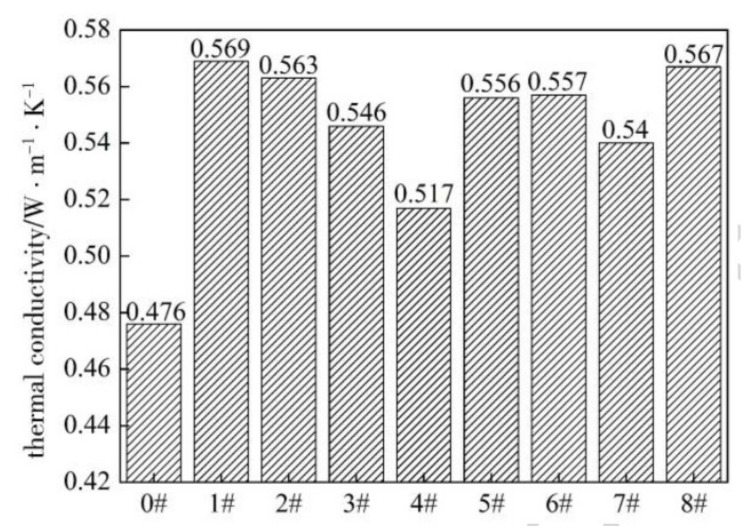
Trend of the thermal conductivity for neat (0#) and differently surface-modified (1#-8#) graphite/NR composites [148]. Copyright © 2021 Elsevier B.V. All rights reserved.

NR nanocomposites enclosing polyacrylate-coated graphite fillers displayed higher λ values compared with those with neat graphite (0#), with a maximum of 0.569 Wm^−1^K^−1^ occurring for sample 1#, which was obtained by a 1:1 ratio between methyl methacrylate and *n*-butyl acrylate (Figure 6). The authors claim that surface modification improves the compatibility and the dispersion of graphite particles in the rubber matrix, thereby hindering phonon scattering effects at the interface and leading to high thermal conductivity.

These results, complemented by the increased crosslink density, extended scorch time, and short vulcanization time of the composites, encourage the application of modified graphite for promoting heat transport and dissipation in rubber composites for tires.

In summary, carbon-based fillers constitute a class of materials widely used for the thermal management in rubber nanocomposites because of their high thermal and electrical conductivities in addition to their light weight and generally affordable cost. However, their electrical conductivity may discourage their application in thermal interface materials (TIMs), where quick heat dissipation and good electrical insulation are required to prevent short circuiting [1]. Moreover, the unique anisotropic structures of carbon-based thermally conductive fillers entail the necessity of providing either an orientation or networking inside the rubber matrix to achieve satisfactory λ enhancements, with the consequent risk of peeling and delamination phenomena that, for some particular applications such as tires, induce severe drawbacks.

A promising alternative to carbonaceous fillers is represented by ceramics such as Al_2_O_3_, BN, and ZnO, which display lower λ values (see Table 1), but are basically better insulators and whose structure, particle shape and size, and surface features can be tailored to readily build up thermally conductive pathways within the rubber matrix. Moreover, they can also be utilized in combination with carbon-based thermally conductive fillers in hybrid systems, supplying a decisive upgrade in the heat transfer performance.

Hereafter, a survey of their characteristics and applications in rubber nanocomposites with advantages, downsides, and some perspectives for use in tire formulation is reported.

### 3.2. Inorganic Filler Systems

#### 3.2.1. Alumina

Al_2_O_3_ is a widely used filler in the field of rubber nanocomposites, endowing improved thermal conductivity as well as good mechanical and electrical properties [149,150]. As reported in Table 1, this material has a thermal conductivity ranging from 30 to 38 W m^−1^ K^−1^, depending on the crystalline phase, which surely are not the best λ values for ceramic fillers. However, alumina is cheap, non-toxic, not particularly harmful, and can easily be obtained in various sizes and shapes, prompting its application in several rubber formulations.

Generally, small particle sizes and high Al_2_O_3_ loadings are needed to achieve the desired enhancement in the thermal conductivity of rubber nanocomposites [151,152], causing remarkable drawbacks in terms of an increase in viscosity, agglomerations of filler, and a reduction in the mechanical properties.

**Figure 7:**
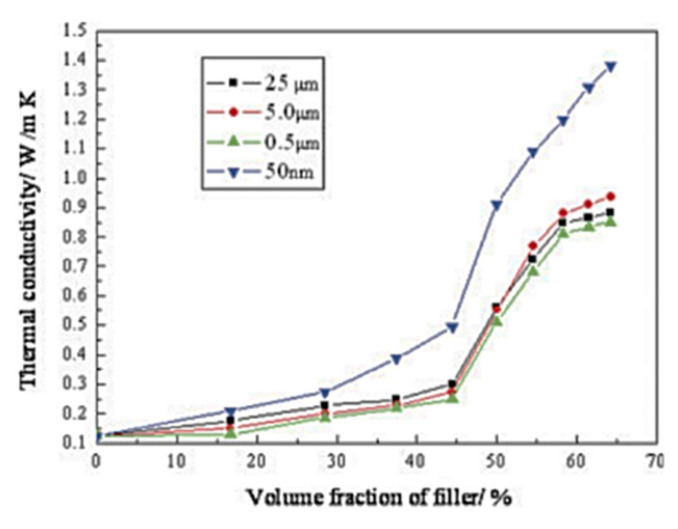
Effect of Al_2_O_3_ particle size on the thermal conductivity of silicone rubber nanocomposites as a function of filler volume fraction [153]. Copyright© 2021 Wiley Periodicals, Inc. All rights reserved.

This is clearly outlined in the study of Zhou et al. [153], where the effect of alumina fillers with different particle sizes on both the thermal conductivity and mechanical properties of SR nanocomposites was explored. In detail, the thermal transport performance imparted by micrometric β-alumina with different particle sizes (0.5–5.0 and 25 μm) and that imparted by nanometric α-alumina (Figure 7) surface functionalized with 3-methacryloyloxypropyltrimethoxysilane, as a compatibilizing coupling agent, were compared. It turned out that, at volume fractions above 50%, SR enclosing nanometric Al_2_O_3_ particles displayed the highest λ values. This result was explained by the authors considering the high packing density achievable with these particles, which provides thermally conductive pathways in the composite. However, the authors also reported that, at such high filler concentrations, the tensile strength and elongation at break of the composites remarkably decreased, raising the issue of accomplishing enhanced thermal conductivity and remarkable mechanical properties simultaneously.

Trying to take on this challenge, Ouyang et al. [154] utilized nano-sized α-Al_2_O_3_ spheres, obtained by high-frequency thermal plasma, to produce Al_2_O_3_/SR nanocomposites displaying both excellent thermal conductivity and satisfactory mechanical properties.

**Figure 8:**
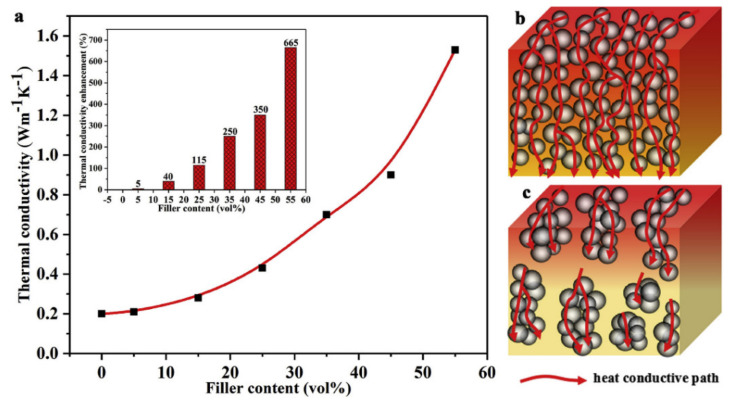
(**a**) Thermal conductivity of the SR composites filled with different contents of Al_2_O_3_ nanospheres and heat flow models of composites with good dispersion (**b**) and poor dispersion (**c**) [154]. Copyright©2020 Elsevier Ltd. All rights reserved.

Taking advantage of the peculiar properties of spherical fillers with smooth surfaces, which do not exhibit edges or corners that can cause significant filler–matrix and filler–filler friction effects during mixing or compounding [11,155], SR nanocomposites with high alumina percolation thresholds (~50–60 *v*/*v*%) were fabricated. The resulting material produced not only an enhancement in λ by 6.65 times, compared with neat SR (Figure 8), but also a remarkable improvement in mechanical properties, such as tensile strength, modulus, and fracture toughness. These results point out the importance of the dispersion and suitable compatibility between alumina fillers and the rubber matrix, key factors in the formation of networks that promote thermal diffusion and lower the transfer of stress.

With the same goal, Song et al. [150] developed α-Al_2_O_3_/SR composites by foaming and subsequent infiltration processes, where alumina particles constituted a thermally conductive 3D network, greatly improving the λ of the final material, without compromising their high electrical insulation. In particular, the peculiar 3D structure promotes the generation of thermally conductive pathways, which impart thermal conductivities significantly higher than those of SR matrices (usually displaying a λ value of 0.1320 W m^−1^ K^−1^) and of α-Al_2_O_3_/SR composites with randomly dispersed filler particles (R-Al_2_O_3_/SR), even at relatively low filler loadings (<25% m/m). Thus, the proposed strategy may represent a step forward in the preparation of high-performance rubber composites for heat dissipation with low filler volume fractions.

**Figure 9:**
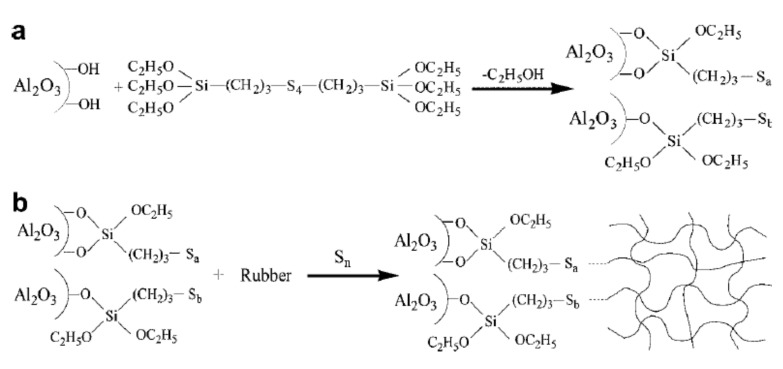
(**a**) In situ surface silanization of α-Al_2_O_3_ nanoparticles; (**b**) expected reaction between functionalized α-Al_2_O_3_ and rubber chains during the vulcanization process [97]. Copyright © 2021 John Wiley & Sons, Ltd.

Very few reports in the literature focus on the exploitation of Al_2_O_3_ for tire applications. Wang et al. [97] utilized α-Al_2_O_3_ for the fabrication of thermally conductive ethylene propylene diene monomer (EPDM) nanocomposites. The influence of the filler volume fraction, the in situ surface functionalization with a silane coupling agent commonly utilized in tire formulations (Si69, bis-(3-triethoxy silylpropyl)-tetrasulfide, Figure 9), and an α-Al_2_O_3_ pre-treatment with stearic acid (SA) on the heat transport and mechanical features was investigated.

**Figure 10:**
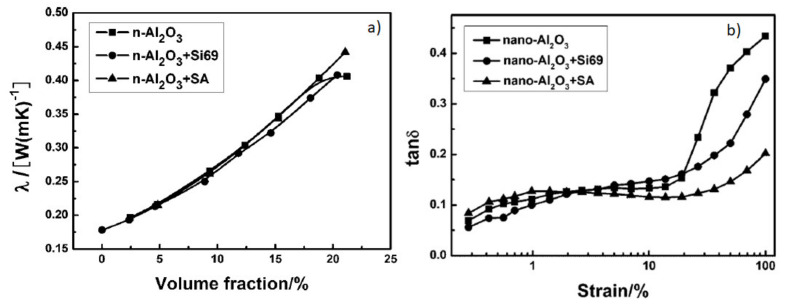
(**a**) Trend of the thermal conductivity (λ) of EPDM nanocomposites as a function of α-Al_2_O_3_ filler volume fraction; (**b**) tan δ of α-Al_2_O_3_/EPDM nanocomposites vs. strain at a filler loading of 149 phr [97]. Copyright © 2021 John Wiley & Sons, Ltd. All rights reserved.

The results evidence that the incorporation of high volume fractions of alumina can effectively upgrade the λ in EPDM nanocomposites, notwithstanding the surface chemical modification of the nanoparticles (Figure 10a). However, the high filler loading (149 phr) affects the tan δ values, which increase as a function of the applied strain, particularly for the naked α-Al_2_O_3_ nanoparticles (Figure 10b).

Besides being an indicator of the dynamic mechanical properties, tan δ can also be considered as an indirect measure of the dynamic heat build-up in rubber composites, which, in general, is associated with the friction phenomena occurring among rubber–rubber, filler–rubber, and filler–filler composites [156,157]. In principle, a continuous filler network hinders filler–filler friction, while enhanced interactions at the hybrid interface should decrease the relative slippage and the filler–rubber friction. Thus, the lower tan δ of rubber nanocomposites enclosing surface-modified Al_2_O_3_ nanoparticles (Figure 10b) can be regarded as the result of improved filler–rubber interactions, which may promote the generation of a more continuous filler network and, in turn, a better efficiency in the heat dissipation.

**Figure 11:**
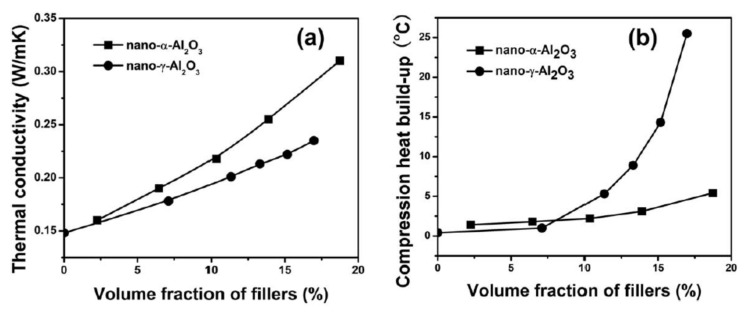
Trend of (**a**) thermal conductivity and (**b**) heat build-up properties of NR composites filled with α-Al_2_O_3_ and γ-Al_2_O_3_ nanoparticles [55]. Copyright© 2021 Society of Plastics Engineers. All rights reserved.

The same authors extended the use of α and γ-Al_2_O_3_ fillers to the fabrication of NR nanocomposites [55] for tire applications. They observed that γ-Al_2_O_3_ imparts superior mechanical properties to NR compared with α-Al_2_O_3_, with a maximum tensile strength 31.9 MPa at 50 phr. Instead, α-Al_2_O_3_ makes the difference in the enhancement of the thermal conductivity (Figure 11a).

This has been attributed on one hand to the lower intrinsic λ and on the other hand to the smaller particle size and higher specific surface area of γ-Al_2_O_3_ nanoparticles, which may cause worse dispersion, uncontrolled aggregation, and more filler–filler friction, leading to the higher heat build-up retrieved for γ-Al_2_O_3_/NR nanocomposites (Figure 11b). Notwithstanding, to achieve satisfactory λ values, high α-Al_2_O_3_ loadings are required (above 100 phr), which ultimately will deteriorate the dynamic mechanical properties of the nanocomposites.

In summary, the above-described studies demonstrate that low-cost and highly abundant alumina fillers are attractive materials for improving the thermal conductivity properties of rubber composites and for large-scale applications, such as tire technology. In this specific frame, though some benefits both in the reinforcing action and λ enhancement can be achieved by modulating the filler’s crystalline phase, morphology, and surface functionalization, the reduction of the Al_2_O_3_ loading still constitutes a challenging point, which may be overcome through its combination with other highly thermally conductive anisotropic fillers (see Section 4).

#### 3.2.2. Boron Nitride

BN is a promising thermally conductive filler thanks to its high intrinsic λ, lubrication, improved thermal and chemical resistance, high electrical resistivity, low density, and moderate cost [158,159,160,161,162]. The hexagonal form of BN (h-BN) displays a graphite-like structure and is sometimes called “white graphene” [25,163]. h-BN is the most stable and soft BN polymorph, and can easily be exfoliated to a single or a few atomic layer sheets [164]. In particular, h-BN nanosheets (BNNSs) have one of the highest in-plane thermal conductivity coefficients (around 2000 Wm^−1^K^−1^), whereas that of bulky hexagonal boron nitride reaches approximately 400 Wm^−1^K^−1^, and the λ value increases as the exfoliation degree increases [164,165].

h-BN fillers have been introduced into a variety of polymer matrices, including rubber, imparting a high λ value and remarkable electrical resistivity [25,166,167,168,169], which are suitable characteristics for the thermal management of electronic devices [170].

**Figure 12:**
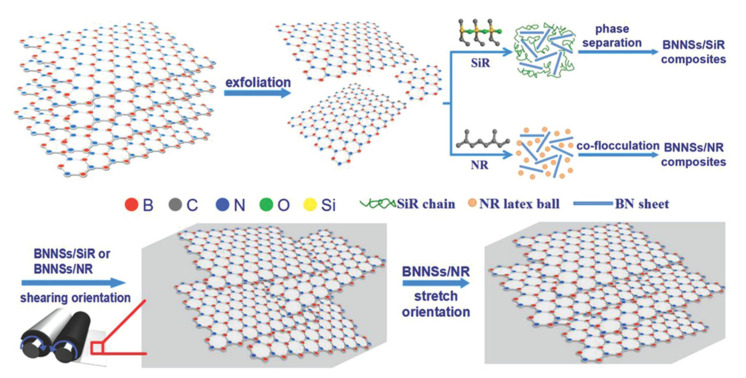
Scheme of the preparation of BNNSs/SiR and BNNSs/NR nanocomposites [171]. Copyright© 2014 WILEY-VCH Verlag GmbH & Co. KGaA, Weinheim. All rights reserved.

Kuang et al. [171] exploited BNNSs to fabricate silicone rubber (SiR) and NR nanocomposites with high thermal conductivity. The bulky h-BN was first exfoliated via ultrasonication to obtain BNNSs, which were successively compounded in solution with SiR and NR latex. BNNSs/SiR and BNNSs/NR nanocomposites with a homogeneous dispersion of filler were obtained after phase separation and co-flocculation procedures, respectively (Figure 12, top). In order to take advantage of the high in-plane λ of BNNSs, filler orientation in BNNSs/SiR and BNNSs/NR compounds was achieved by oscillatory shearing on a two-roll mill, followed by vulcanization, which reduces the molecular mobility and stabilizes the BNNSs’ orientation (Figure 12, bottom).

**Figure 13:**
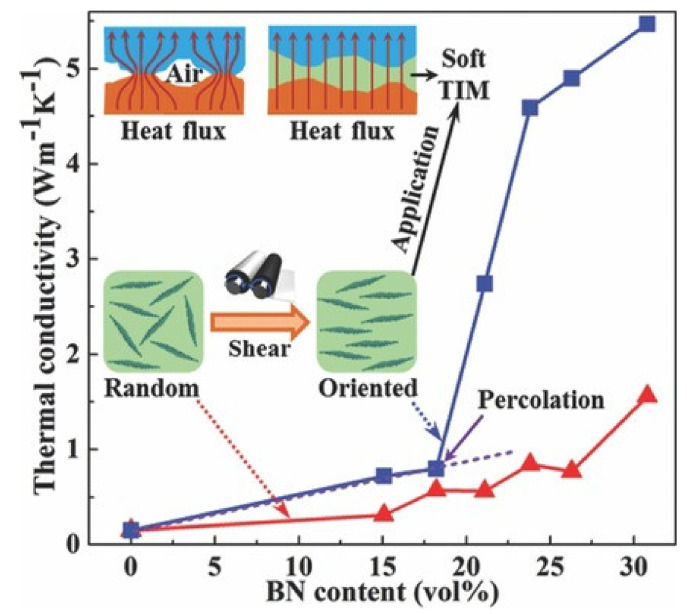
Thermal conductivity of BNNSs/SiR and BNNSs/NR nanocomposites as a function of filler loading [171]. Copyright© 2021 WILEY-VCH Verlag GmbH & Co. KGaA, Weinheim. All rights reserved.

The results show better thermal conductivity performance for BNNSs/SiR compared with BNNSs/NR after a simple oscillatory shear test, especially when the filler loading exceeds 17.5 *v*/*v*% (Figure 13).

However, a relatively high interfacial thermal resistance generally occurs at the hybrid interface between h-BN platelets and the polymer matrix, hindering the thermal transport [172]. To overcome this drawback, BN surface modification with silane coupling agents [173,174,175], via in situ polymerization [167] and polymer grafting [176,177], has been attempted.

However, though these covalent approaches can supply strong interfacial adhesion between the filler and the matrix, with a consequent reduction in the thermal resistance and an improvement in mechanical properties, they may lead to damage to the original BN structure, thereby reducing the inherent λ [178].

**Figure 14:**
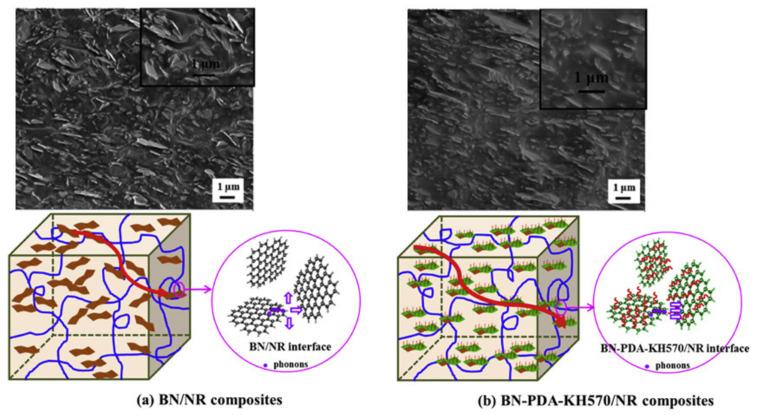
SEM images and heat dissipation/transport models for (**a**) BN/NR and (**b**) BN-PDA-KH570/NR nanocomposites. Adapted from [179]. Copyright© 2021 Elsevier Ltd. All rights reserved.

Alternatively, Yang et al. [179] proposed a combination of covalent and non-covalent functionalization of h-BN platelets by a preliminary modification with polydopamine (PDA) and subsequent grafting with γ-methacryloxypropyl trimethoxy silane (KH570). The BN and BN-PDA-KH570 platelets were blended with NR and, after vulcanization, BN/NR and BN-PDA-KH570/NR nanocomposites were obtained. In the BN/NR composites, the presence of randomly oriented aggregates and of fracture surfaces between the BN platelets and the NR matrix was evident (Figure 14a), indicating weak interfacial interactions of BN/NR composites. Interestingly, the surface modification with PDA improved the compatibilization, while the reactivity of the double bonds of KH570 with the rubber chains enhanced both their dispersion and interfacial interaction, leading to the generation of oriented platelets homogeneously distributed within the NR matrix (Figure 14b).

**Figure 15:**
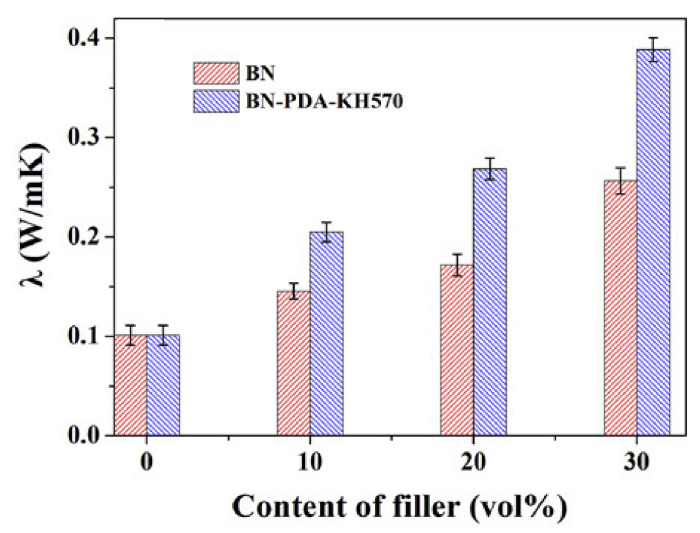
λ values retrieved for bare NR, BN/NR nanocomposites, and BN-PDA-KH570/NR nanocomposites [179]. Copyright© 2021 Elsevier Ltd. All rights reserved.

These unique features promote the generation of efficient thermally conductive pathways in BN-PDA-KH570/NR nanocomposites (models in Figure 14), which results in higher thermal conductivity compared with BN/NR (Figure 15).

Similar outcomes have been obtained in NBR [180], corroborating the decisive role of the filler–rubber interaction at the hybrid interface on the thermal conductivity and holding the promising application of BN as a thermal management material for electronic devices.

Currently, most of the preparation methods for polymer/BNNSs composites not suitable for large-scale production and are directed toward the thermal management materials of electronic devices.

**Figure 16:**
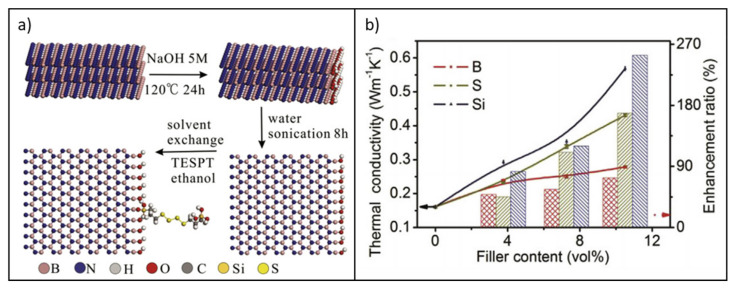
(**a**) Scheme of the preparation procedure for BNNS slurry; (**b**) thermal conductivity and enhancement ratio of SBR/BN (B), SBR/BNNSs (S), and SBR/Si-BNNSs (Si) composites at different filler loadings. Adapted from [181]. Copyright© 2021 Elsevier Ltd. All rights reserved.

However, Wu et al. [181] recently proposed a cost-effective and environmentally friendly water/ethanol-based approach to the preparation of BNNSs/SBR nanocomposites by liquid exfoliation/solvent exchange. The slurry was compounded with SBR in the presence of Bis-(γ-triethoxysilylpropyl)-tetrasulfide (TESPT), a typical silane coupling agent employed in rubber composites for tires, in order to provide a suitable filler compatibilization with the rubber matrix (Figure 16a).

In situ modification with TESPT not only upgraded the mechanical properties of the composites (Si-BNNSs/NR) but, more significantly, greatly improved the thermal conductivity properties. In fact, with a loading of just 10.5 *v/v*% of Si-BNNSs filler, λ was increased by 253% compared with the neat SBR (Figure 16b).

This approach can endow an effective transfer of the dispersion state of BNNSs achieved in slurry to the polymer matrix, allowing for a significant increase in mechanical properties as well as in thermal transport, even at relatively low filler loadings.

Regarding on the potential exploitation of BN in thermally conductive rubber composites for tires, very few reports in the literature envisage this application. The main limitations lie in the high surface energy and amphiphobic nature of BN nanosheets, which result in a poor dispersion ability, disordered and unwanted aggregation, and high thermal resistance at the hybrid interface.

**Figure 17:**
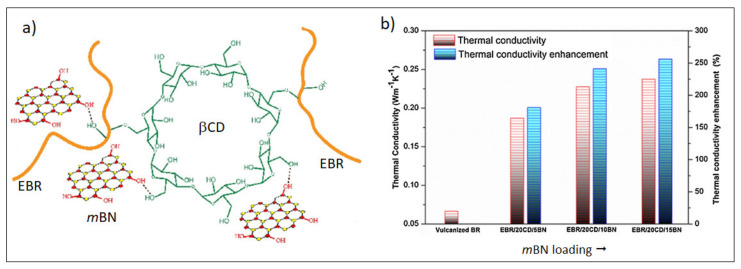
(**a**) Mechanism of the generation of EBR/βCD/*m*BN nanocomposites; (**b**) Trend of the thermal conductivity for vulcanized BR and EBR-based nanocomposites at 25 °C. Adapted from [182]. Copyright© 2021 Elsevier Ltd. All rights reserved.

Taking on these tasks, Yang et al. [182] proposed the incorporation by solution blending in epoxy BR (EBR) of hydroxylated h-BN (*m*BN) modified at the surface with biological β-cyclodextrin (βCD), which acts as a non-toxic interfacial cross-linking agent, promoting the dispersion of *m*BN by hydrogen bond interactions (Figure 17a).

The resulting nanocomposites displayed an improved storage modulus, *T*_g_, and thermal stability along with an increase in filler content. Interestingly, the λ value of EBR/βCD/*m*BN nanocomposites comprising only 4 wt.% of *m*BN was remarkably higher than that of vulcanized BR, supporting the potentiality of boron nitride for the thermal management of rubber compounds utilized in tire tread (Figure 17b).

In conclusion, BN, thanks to its high thermal conductivity, structural stability, good mechanical properties, and antioxidation ability, is an attractive material for enhancing the functional properties of rubber composites. However, as with other 2D fillers such as graphene, the structural anisotropy of BNNSs is critical and accounts for their orientation in the polymer matrix, which determines the final λ enhancement. Moreover, a suitable balance between BNNS dispersion and networking represents an essential matter to be tailored by surface functionalization in order to minimize the interfacial thermal resistance and to promote the generation of thermally conductive pathways.

#### 3.2.3. Zinc Oxide

ZnO, a semiconductor widely employed in catalysis [183], optoelectronics [184], sensors [185,186], and several formulation products such as cosmetics [187], sunscreens [188,189], and polymer composites [190], is also the most efficient and used activator for the vulcanization process: more than 50% of the global annual ZnO production is exploited for rubber manufacturing [191], in which tires constitute the principal product [192].

Meanwhile, the fairly good λ value (~60 Wm^−1^K^−1^) also makes ZnO an attractive material for tailoring the thermal management and heat dissipation of polymers [27,95,193,194]. However, very few examples are present in the literature regarding its application in rubber nanocomposites with these targets and as a single filler.

Wang et al. [195] prepared ZnO/EPDM rubber nanocomposites and studied their thermal conduction and mechanical properties in comparison to traditional reinforcing fillers, such as carbon black and SiO_2_ nanoparticles. The influence of ZnO surface functionalization with the bis-(3-thiethoxy silylpropyl)-tetrasufide (Si69) silane coupling agent on the thermal transport and on the static and dynamic mechanical features was also investigated.

**Figure 18:**
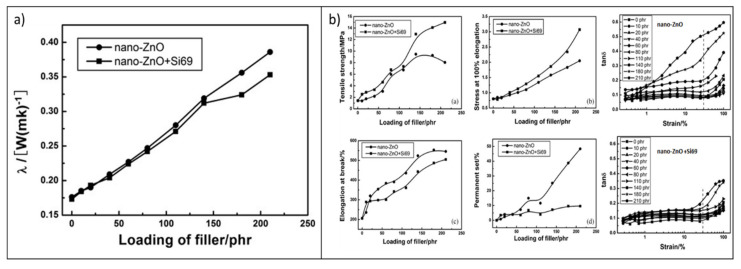
(**a**) Trend of the thermal conductivity of EPDM nanocomposites as a function of bare (nano-ZnO) and surface-functionalized ZnO (nano-ZnO+Si69) nanoparticles; (**b**) summary of the main static and dynamic mechanical properties of ZnO/EPDM rubber nanocomposites. Adapted from [195]. Copyright© 2021 Wiley Periodicals, Inc. All rights reserved.

The results indicate that a significant enhancement in the λ can be achieved only at very high filler loadings (above 120 phr), regardless of the filler silanization (Figure 18a), which instead has much more impact on the mechanical and dissipation properties of the compounds. In fact, surface modification with Si69 improves the filler compatibilization and distribution within the rubber matrix, upgrading the static and dynamic mechanical properties (Figure 18b). Finally, compared with carbon black N330 and silica nanoparticles, EPDM nanocomposites enclosing surface-silanized ZnO nanoparticles display better performances both in terms of thermal conductivity and reinforcing properties, suggesting the suitability of this filler system for the fabrication of elastomer products working under dynamic conditions with a longer expected service life.

**Figure 19:**
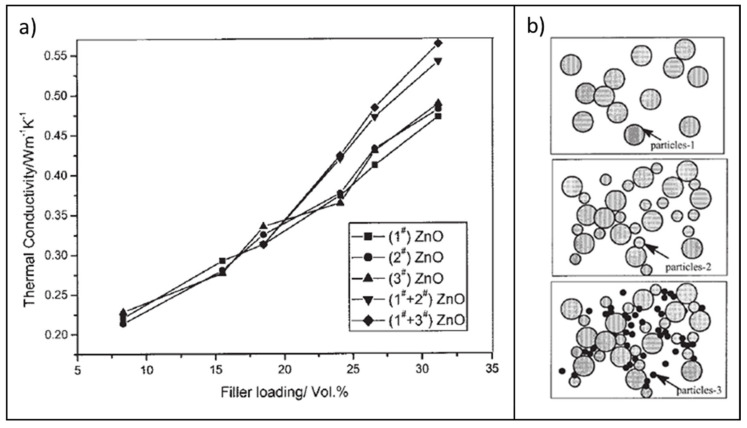
(**a**) Trend of the thermal conductivity in ZnO/SR nanocomposites as a function of filler loading and size (1^#^ZnO, 2^#^ZnO, 3^#^ZnO); (**b**) Sketch of the thermal conduction models for silicone rubber enclosing a single filler or ZnO particles with different sizes. Adapted from [26]. Copyright© 2018 Wiley Periodicals, Inc. All rights reserved.

In a similar context, Mu et al. [26] prepared ZnO/silicone-rubber nanocomposites and studied the effects of filler loadings and particle sizes on the thermal conductivity. Experimental results revealed that λ gradually increased with the volume fraction of ZnO, particularly when submicrometric filler particles (average particle size ~0.15 μm) were utilized (Figure 19a). Noticeably, it was observed that blending ZnO particles with different sizes induces a more remarkable enhancement of the thermal conductivity, even at relatively low loadings (Figure 19b).

The effect was explained by considering that, when differently sized fillers are simultaneously introduced within the polymer matrix, higher packing fractions can be achieved, leading to the generation of more continuous thermally conductive pathways that boost the thermal transport throughout the whole composite (Figure 19b). This is actually the operating principle of hybrid fillers, which will be described in detail for several Al_2_O_3_, BN, and ZnO systems in the next section.

To sum up, ZnO has great potential for thermal management and heat dissipation in rubber composites. This entails the optimization of the micro/nano structure of the material as well as suitable surface functionalization approaches, which may enable its application in tire formulations as a multifunctional filler imparting enhanced reinforcing, vulcanizing, and thermal conductivity properties [196].

## 4. Hybrid Fillers: A New Frontier for the Thermal Management of Rubber Nanocomposites

Hybrid fillers are mixtures of different kinds of thermally conductive fillers or mixtures of the same material with different structural or morphological features. The main reason that prompted the development and the use of hybrid fillers is the difficulty of achieving the theoretically high *λ* value for polymer composites by the addition of a single thermally conductive filler, mainly due to phonon scattering phenomena induced by defects, interfaces, and by the difficulty of processing related to high filler loadings [14]. Instead, hybrid fillers can improve the dispersion and packing fraction, thereby improving the bridge among adjacent materials and promoting the generation of thermally conductive pathways. Additionally, a lower percolation threshold can be attained, which is important to reduce cost, to facilitate the processing of composites, and to preserve mechanical properties [16,78].

Commonly, binary hybrid fillers are employed, such as SiC or BN with a different morphology [42,197], carbonaceous/inorganic systems such as CNT/Al_2_O_3_ [193] and graphene/Ag [198], and carbonaceous/carbonaceous or inorganic/inorganic systems such as graphene/CNT [199,200] and BN/Al_2_O_3_ [201], respectively. However, combinations of three distinct conductive materials can also be exploited [202].

Concerning rubber nanocomposites and focusing on inorganic/inorganic and carbonaceous/inorganic systems, several examples can be found in the recent literature.

For instance, Li et al. [203] reported on the preparation of SR nanocomposites with enhanced thermal conductivity enclosing both spherical and tetrapod-shaped ZnO nanoparticles (labeled ZnOs and ZnOw, respectively), constituting a hybrid filler. ZnOs were also surface modified with a silane coupling agent to provide a suitable compatibilization with the SR matrix (i.e., m-ZnO nanoparticles).

**Figure 20:**
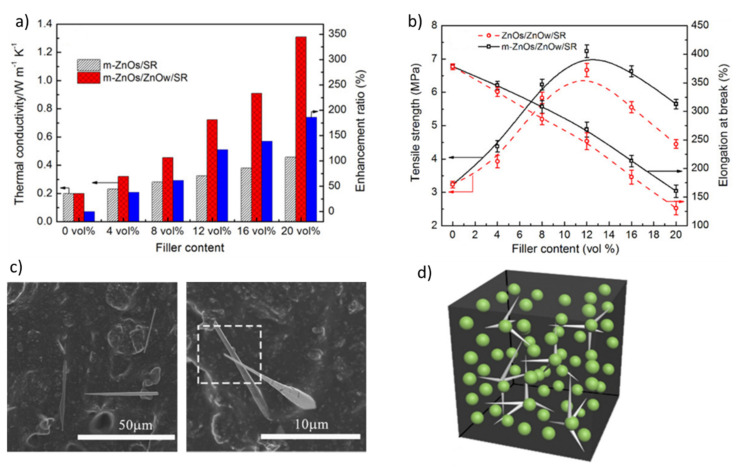
(**a**) Comparison of thermal conductivity between m-ZnOs/SR and m-ZnOs/ZnOw/SR nanocomposites at different filler volume fractions; (**b**) Tensile strength and elongation at break of the ZnOs/ZnOw/SR and m-ZnOs/ZnOw/SR nanocomposites; (**c**) Cross-section morphologies of the m-ZnOs/ZnOw/SR nanocomposites enclosing 8 *v*/*v*% of hybrid filler; (**d**) Schematic illustration of the thermally conducting network in the SR composite filled with m-ZnOs and m-ZnOs/ZnOw. Adapted from [203]. Copyright© 2018 Wiley Periodicals, Inc. All rights reserved.

Results show that the thermal conductivity of the m-ZnOs/ZnOw/SR nanocomposites increases as the hybrid filler volume fraction increases and significant enhancements compared with the neat SR matrix can be attained, even at low loadings (Figure 20a). Noticeably, the substitution of ZnOs with ZnOw not only increases the λ value (Figure 20b), but results in improved mechanical properties, namely in terms of tensile strength. These outcomes have been associated with the ability of tetrapod-shaped ZnO structures to form bridges among m-ZnOs nanoparticles, leading to the generation of a 3D thermally conductive network within the rubber matrix, which fosters phonon transfer and enhances the thermal conductivity (Figure 20c,d). In light of these considerations, SR nanocomposites with hybrid ZnO filler appear to be promising candidates for applications in thermally conductive and electrically insulating materials.

**Figure 21:**
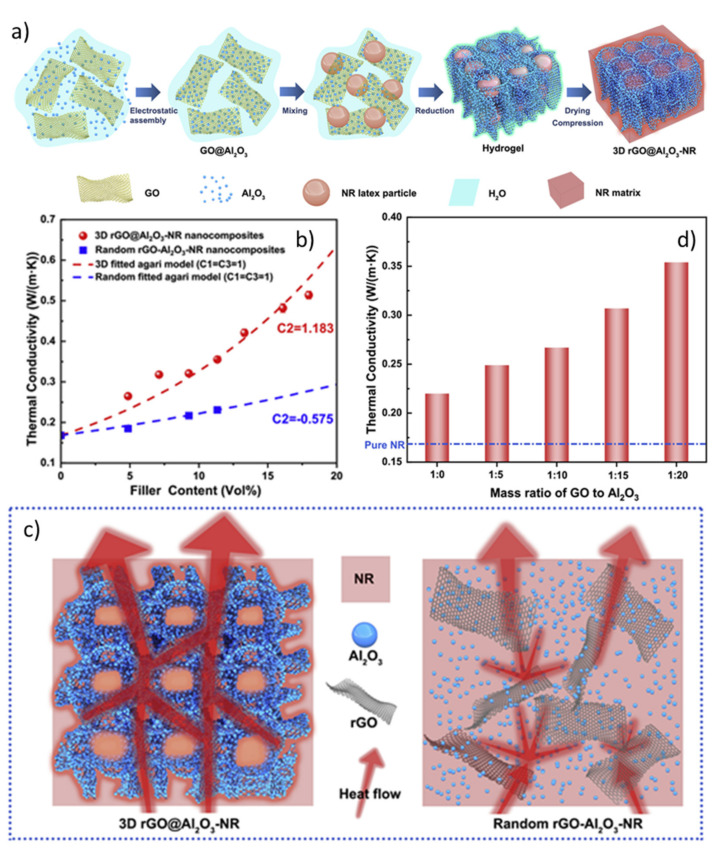
(**a**) Scheme of the procedure for the fabrication of 3D rGO@Al_2_O_3_-NR nanocomposites; (**b**) Trend of the thermal conductivity for 3D rGO@Al_2_O_3_-NR and Random rGO-Al_2_O_3_-NR nanocomposites as a function of filler loading; (**c**) Illustration of the heat transfer mechanism in both compounds; (**d**) thermal conductivity of 3D rGO@Al_2_O_3_-NR nanocomposites vs. the rGO/Al_2_O_3_ mass ratio. Adapted from [204]. Copyright© 2019 Published by Elsevier Ltd. All rights reserved.

Li et al. [204] fabricated nanocomposites comprising rGO nanosheets decorated on the surface with γ-Al_2_O_3_ nanoparticles. The hybrid rGO@Al_2_O_3_ filler was obtained by electrostatic self-assembly and then mixed by solution-blending with NR latex. Subsequently, the mixture underwent reduction to generate a hybrid hydrogel, followed by drying and compression to obtain 3D rGO@Al_2_O_3_-NR composites (Figure 21a).

The thermal conductivity properties of the produced nanocomposites were compared with those of compounds fabricated by randomly mixing rGO and γ-Al_2_O_3_ (namely Random rGO-Al_2_O_3_-NR).

The 3D rGO@Al_2_O_3_-NR exhibited a higher λ than that of the nanocomposites prepared by a conventional method at the same filler loading (Figure 21b). According to fracture surface SEM images, this can be attributed to the generation of a 3D interconnected structure, which enables efficient heat transfer paths, thus upgrading the thermal conductivity of the composite (Figure 21c).

More interestingly, the authors also observed that λ increases (Figure 21d) upon increasing the alumina coverage on rGO, confirming the ability of hybrid fillers to promote the heat transfer and suggesting the possibility of tailoring the thermal management of the material simply by changing the Al_2_O_3_ proportions.

**Figure 22:**
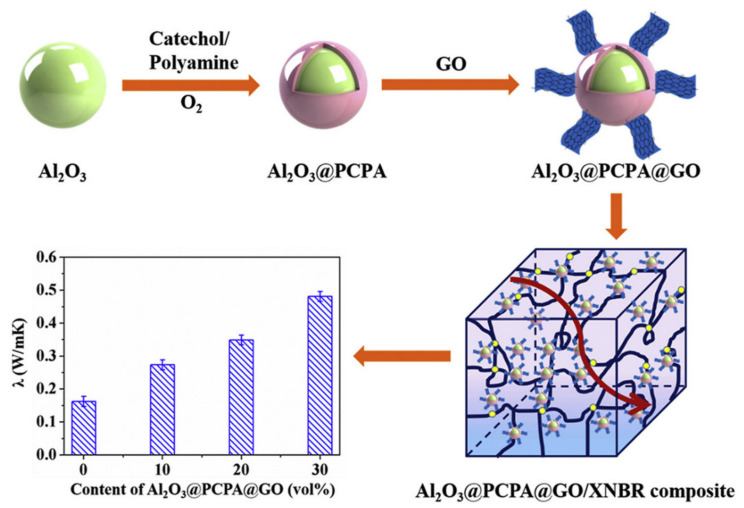
Schematic representation of preparation procedure, thermal dissipation/transfer model, and thermal conductivity properties of Al_2_O_3_@PCPA@GO/XNBR composites [205]. Copyright© 2020 Published by Elsevier Ltd. All rights reserved.

Yang et al. [205] fabricated alumina@poly(catechol-polyamine)@graphene oxide (denoted Al_2_O_3_@PCPA@GO) multilayer core–shell hybrid fillers, which were then utilized to improve the thermal conductivity properties of carboxyl NBR (XNBR) latex (Figure 22).

The combination of Al_2_O_3_ and GO leads to the formation of a “dual network” structure composed by alumina “islands” and GO “bridges”, respectively, which boosts both the λ value and the mechanical properties of the composite (Figure 22). Furthermore, an improvement in the dielectric constant and insulation properties was attained for the Al_2_O_3_@PCPA@GO/XNBR nanocomposites, envisaging their potential application as thermally conductive materials for electronic equipment.

The thermal conductivity of rubber nanocomposites can be also improved by modifying ceramics or carbonaceous fillers with metal particles [206,207]. In this context, Al_2_O_3_-PDA-Ag hybrid nanoparticles were synthesized and then blended with NR to yield elastomer composites [206]. The surface functionalization with PDA provides enhanced interfacial interaction between the alumina and the NR matrix while the Ag nanoparticles, due their high intrinsic λ, remarkably upgrade the thermal conductivity of the hybrid filler, inducing efficient thermal transport in the resulting nanocomposites, even at a relatively low filler loading (10 *v*/*v*%) [206].

Incorporation of hybrid fillers has been accepted as a promising strategy to prepare high-performance rubber nanocomposites while complying with industrial requirements in the field of tire manufacturing.

While the effect of hybrid filler systems, such as silica/carbon black [208,209] and modified layered silicate/carbon black [210], on the mechanical properties of SBR-based tire tread formulations has been well addressed, their exploitation for the achievement of an optimum balance between dynamic mechanical and heat dissipation properties remains a challenge [211].

**Figure 23:**
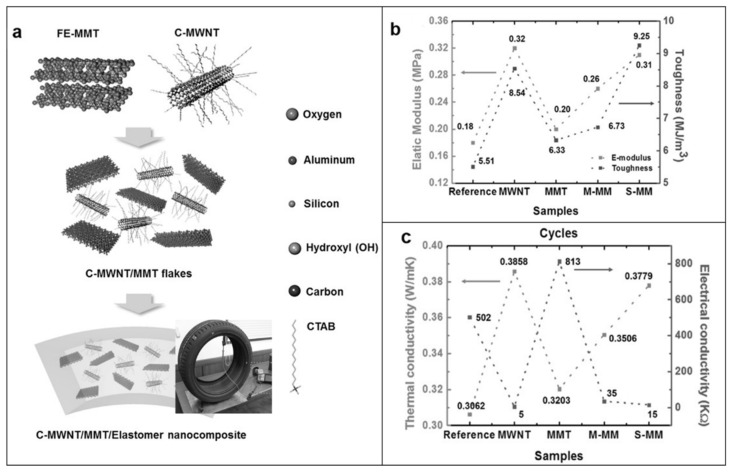
(**a**) Scheme of the nanocomposite’s preparation; (**b**) Elastic modulus and toughness of the elastomer nanocomposites enclosing neat fillers (MWNT, MMT), hybrid filler (S-MM), and mixed fillers (M-MM); (**c**) Thermal and electrical conductivities of the elastomer nanocomposites enclosing different fillers. Adapted from [212]. Copyright© 2016 Wiley Periodicals, Inc. All rights reserved.

To resolve this issue, Song et al. [212] prepared hybrid fillers composed of hydroxyl-functionalized exfoliated montmorillonite (FE-MMT) and cetyltrimethylammonium bromide-modified multiwall nanotubes (C-MWNTs). These materials were subsequently co-coagulated with SBR latex to obtain elastomeric nanocomposites (labeled S-MM, Figure 23a). Nanocomposites fabricated by simply mixing FE-MMT and C-MWNT with SBR (M-MM) were also considered for comparison purposes. A remarkable improvement in both the mechanical (Figure 23b) and thermal conductivity properties (Figure 23c) of S-MM composites was observed even at low hybrid filler loadings (e.g., 5 phr), particularly when compared with M-MM and with the compound enclosing neat C-MWNT and FE-MMT fillers.

These superior properties were exploited in the formulation of the tread of a pneumatic tire, showing enhanced wet grip and low-rolling resistance. The results, besides highlighting the synergistic action of clays and CNTs in the hybrid filler, suggest that its utilization is highly promising for the preparation of rubber composites with low loading levels and remarkable thermal conductive and mechanical performance.

More recently, the same authors prepared hybrid fillers based on silica nanoparticles decorating graphene nanosheets and investigated them for potential use in the preparation of SBR nanocomposites for tires [213,214].

**Figure 24:**
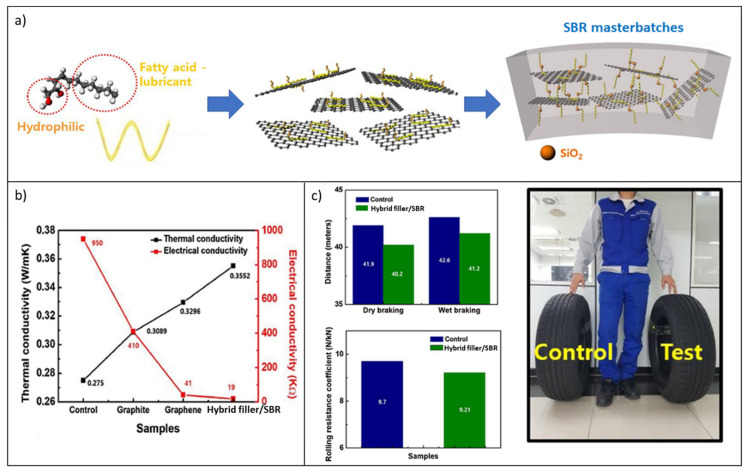
(**a**) General scheme of the Hybrid/SBR nanocomposites’ preparation; (**b**) Thermal and electrical conductivities of the SBR nanocomposites with different filler materials; (**c**) Characterization of tread compounds (control and those made with nanocomposites enclosing hybrid fillers). Modified according to [213]. Copyright© 2018 Published by Elsevier B.V. on behalf of The Korean Society of Industrial and Engineering Chemistry. All rights reserved.

In particular, in order to meet the ever-growing environmental and safety requirements for rubber formulations, zinc-free eco-friendly coupling agents were utilized to provide graphene compatibilization with the polymer matrix and enhanced interfacial interaction with SiO_2_ nanoparticles. Masterbatches of SBR/graphene silica nanohybrids (Hybrid filler/SBR) were obtained by a fast, inexpensive, scalable, and versatile latex co-coagulation approach (Figure 24a). Their mechanical properties and thermal and electrical conductivities, even at low filler loadings (e.g., 5 phr), were significantly higher than those of similar SBR nanocomposites enclosing silica decorating carbon black, graphite, and rGO prepared by the same procedure (Figure 24b). The peculiar characteristics of the reported hybrid filler provide enhanced interfacial adhesion of the filler to the rubber and the generation of a continuous filler network, upgrading the reinforcement and facilitating thermal transport through the matrix.

To substantiate the possible application of these hybrid fillers in “green tires”, the produced master batches were added to silica/SBR-based tread formulations commonly used to manufacture a pneumatic tire. The tread compounds showed lower rolling resistance and highly improved grip, in both dry and wet conditions, compared with a reference compound (control), corroborating the great potential of fillers for tire engineering (Figure 24c).

In summary, hybrid fillers seem to represent the “cutting edge” of the effective thermal management of rubber nanocomposites. In fact, besides supplying higher thermal conductivity, they can also help to lower the viscosity and improve the processability of the composites due to the reduced filler loadings required for attaining bridging effects and, thus, thermally conductive paths. The modulation of the ratio among the different materials constituting the hybrid plays a key role in determining the filler networking and orientation, with remarkable fallouts in the control of the anisotropic/isotropic thermal transport within the matrix. This point is of critical importance for rubber nanocomposites utilized in tires, where a trade-off between high thermal dissipation and hindering of delamination/peeling phenomena is essential for extending the product’s lifetime.

## 5. Conclusions and Perspectives

In this review, we concentrated our endeavors on summarizing the use of inorganic and hybrid fillers for the thermal management of rubber nanocomposites, with a specific focus on the prospective application in tire formulations.

The picture retrieved from the described studies discloses the potentialities of inorganic nanomaterials in upgrading the thermal conductivity of rubber polymers, without hiding some important issues that still limit their utilization, especially in large-scale technologies. In particular, besides the ability to select highly available, low-cost, and environmentally sustainable systems, the identification of experimental approaches for lowering both the filler loading and thermal interfacial resistance remain crucial aspects for their employ as an alternative to carbonaceous fillers.

The most promising avenue encompasses synthesis or modification strategies that improve their aspect ratio and, simultaneously, provide a targeted surface functionalization, assuring remarkable interfacial adhesion with the rubber matrix and the formation of thermally conductive networks. These attempts include the possibility to control the filler orientation for tuning isotropic and anisotropic thermal transport in the rubber matrix, which may also be exploited for imparting Joule-effect-assisted self-healing to the nanocomposites.

In the same direction and notably for tires, a real breakthrough seems to lie in the emerging utilization of hybrid fillers, since one can “take the best” for instance from carbon-based materials (high thermal and electrical conductivity) and inorganic ones (morphology modulation, high packing density, reinforcing ability) to remarkably upgrade the advantages gained by using a single filler.

Finally, though not stressed in the review, theory and modeling are fundamental to accomplish these targets, since the screening of potential compositions and determining the λ value’s dependence on the polymer matrix, filler volume fraction, and morphology may provide valuable inputs for the economical and sustainable design of thermally conductive rubber nanocomposites.

## Figures and Tables

**Table 1:** Thermal conductivities of the carbonaceous and ceramic fillers considered in this review.

Material	λ W m^−1^ K^−1^)	Type	Reference
Single-wall Carbon Nanotubes	3500 (single tube)	Carbon-Based	[128]
Graphene	3080–5150 (in plane)	Carbon-Based	[129]
Graphite	500–1000	Carbon-Based	[130]
Alumina	38–42	Ceramic	[78]
Hexagonal Boron Nitride	in-plane: 400; smakalout of plane: 2	Ceramic	[131]
Zinc Oxide	50	Ceramic	[1]
Silver	417–427	Metal	[132]

## Data Availability

Data available in a publicly accessible repository. The data presented in this study are openly available in the reported references.
